# Interventions to Prevent Potentially Avoidable Hospitalizations: A Mixed Methods Systematic Review

**DOI:** 10.3389/fpubh.2022.898359

**Published:** 2022-07-11

**Authors:** Cecilie Nørby Lyhne, Merete Bjerrum, Anders Hammerich Riis, Marianne Johansson Jørgensen

**Affiliations:** ^1^Department of Public Health, Aarhus University, Aarhus, Denmark; ^2^Department of Research, Horsens Regional Hospital, Horsens, Denmark; ^3^Department of Clinical Medicine, Centre for Clinical Guidelines and Danish Centre of Systematic Reviews, A JBI Centre of Excellence, Aalborg University, Aalborg, Denmark; ^4^Enversion A/S, Aarhus, Denmark

**Keywords:** primary healthcare, health service, prevention, ambulatory care sensitive conditions, preventable hospitalizations, health policy, integrated synthesis, content analysis

## Abstract

**Background:**

The demand for healthcare is increasing due to an aging population, more people living with chronic diseases and medical comorbidities. To manage this demand, political institutions call for action to reduce the potentially avoidable hospitalizations. Quantitative and qualitative aspects should be considered to understand how and why interventions work, and for whom. The aim of this mixed methods systematic review was to identify and synthesize evidence on interventions targeting avoidable hospitalizations from the perspectives of the citizens and the healthcare professionals to improve the preventive healthcare services.

**Methods and Results:**

A mixed methods systematic review was conducted following the JBI methodology using a convergent integrated approach to synthesis. The review protocol was registered in PROSPERO, reg. no. CRD42020134652. A systematic search was undertaken in six databases. In total, 45 articles matched the eligibility criteria, and 25 of these (five qualitative studies and 20 quantitative studies) were found to be of acceptable methodological quality. From the 25 articles, 99 meaning units were extracted. The combined evidence revealed four categories, which were synthesized into two integrated findings: (1) Addressing individual needs through care continuity and coordination prevent avoidable hospitalizations and (2) Recognizing preventive care as an integrated part of the healthcare work to prevent avoidable hospitalizations.

**Conclusions:**

The syntheses highlight the importance of addressing individual needs through continuous and coordinated care practices to prevent avoidable hospitalizations. Engaging healthcare professionals in preventive care work and considering implications for patient safety may be given higher priority. Healthcare administers and policy-makers could support the delivery of preventive care through targeted educational material aimed at healthcare professionals and simple web-based IT platforms for information-sharing across healthcare settings. The findings are an important resource in the development and implementation of interventions to prevent avoidable hospitalizations, and may serve to improve patient safety and quality in preventive healthcare services.

**Systematic Review Registration:** https://www.crd.york.ac.uk/prospero/display_record.php?RecordID=134652, identifier: CRD42020134652.

## Introduction

The aging population, the increase in the number of people living with chronic disease, and the rise in comorbidity expand the demand for healthcare. Thus, efficient interventions are needed to respond to the care needs in the population (1–3). In 2013, eight causes of chronic diseases, including cardiovascular diseases, chronic obstructive pulmonary disease, and diabetes, affected more than 10% of the world population (4, 5). Unplanned hospitalizations are commonly seen in people with chronic disease due to acute complications (6, 7). Preventing these complications, and ultimately hospitalizations due to chronic disease, would benefit the healthcare system in terms of increased healthcare efficiency, but it would also benefit the individual as hospitalization often negatively influences the quality of life (8–10). Approximately 20–35% of all unplanned hospitalizations are thought to be avoidable if complications are handled well in primary care (11, 12).

Researchers and health authorities often use the term “ambulatory care sensitive conditions” (ACSCs) when referring to hospitalizations deemed to be potentially preventable given appropriate care in the community-based healthcare setting (13–15). The term covers various conditions, but it often comprises diabetes, congestive heart failure and chronic obstructive pulmonary disease (11, 15). In this review, ACSCs and potentially avoidable admissions are considered interrelated. Thus, to conform to the use of various definitions in existing articles, potentially avoidable hospitalizations are not restricted to include specific conditions, but these hospitalizations are broadly conceptualized as hospitalizations that might have been prevented by effective intervention in primary care (hereafter: avoidable hospitalizations) (11, 15). The Organization for Economic Co-operation and Development (OECD) has stressed the need for preventive strategies to reduce the number of avoidable hospitalizations, and the OECD calls for action to increase the availability of primary healthcare and to provide more continuous and coordinated care across healthcare settings (16). Accordingly, the World Health Organization (WHO) have responded to needs for continuity and care coordination by presenting a practical framework to guide integrated people-centered health services to better respond to individual needs (17). To target avoidable hospitalizations, research has highlighted the effect of individual-oriented strategies, such as patient education and self-management, and system-oriented strategies, such as tools to improve coordination, both internally in the community healthcare sector and across primary and secondary care (12, 18). Models of evidence-based healthcare practice have been proposed to acknowledge the importance of including individual values and preferences together with the healthcare professionals' clinical expertise alongside the best available evidence (19, 20). Thus, to understand how and why interventions work (or not) and for whom, both quantitative and qualitative aspects should be considered (20, 21).

A preliminary search in PubMed, PROSPERO, and the Cochrane Library revealed that a systematic review on interventions and/or stakeholders' perspectives on interventions targeting avoidable hospitalizations has not yet been conducted. However, reviews on similar thematic topics were identified. A literature review focused on the care of older people by summarizing literature predicting and preventing avoidable hospitalizations (12). A meta-analysis focused on pharmacist-led interventions to reduce unplanned admissions for older people (22), while another systematic review determined the effectiveness and costs of “hospital at home services” for older people (23). Yet, these reviews were unsystematic or narrowed to older people, or they focused on measures of effectiveness and the costs of a specific intervention. To the authors' knowledge, no systematic review exists on interventions targeting avoidable hospitalizations, and why interventions work or not. The aim of this mixed methods systematic review was to identify and synthesize evidence on interventions targeting avoidable hospitalizations from the perspectives of the citizens (age: ≥18 years) and the healthcare professionals to improve the preventive healthcare services.

## Methods

### Design

Mixed methods systematic review methodology was used to grasp the complexity of the aim. The review was conducted in accordance with the JBI methodology for mixed methods systematic reviews following the convergent integrated approach, i.e., assembling quantitative and qualitative evidence to obtain comprehensive knowledge on why and how interventions work based on the citizens' and the healthcare professionals' perspectives (21, 24). The review protocol was registered in PROSPERO, reg. no. CRD42020134652 (25) (Supplementary Material 1). With reference to the protocol, the review question was specified in line with the convergent integrated approach to synthesis. Therefore, reframed from the focus on effectiveness and/or meaningfulness of interventions to focus intervention components, and why interventions work or not. In addition to Scopus, Embase, PubMed, CINAHL, and Cochrane Library, SveMed+ was added in the search strategy bringing it into agreement with the inclusion criteria, which was changed to include studies undertaken in developed countries with universal healthcare (Context). The inclusion criteria regarding the context were specified and accepted in the protocol to enhance the external validity of integrated findings. The reporting of this mixed methods systematic review adheres to the JBI manual for conducting and reporting a JBI mixed methods systematic review using a convergent integrated approach (Supplementary Material 2).

This review was conducted in seven steps: (1) review question, (2) inclusion and exclusion criteria, (3) search strategy, (4) study selection, (5) quality assessment, (6) data extraction, and (7) data synthesis using content analysis (26–29). To enhance the transparency of the integrative review method, an overview of the phases from aim to lines of action is presented in Supplementary Material 3.

The aim was operationalized in one review question: From the perspectives of the citizens (age: ≥18 years) and the healthcare professionals, which intervention components intend to prevent avoidable hospitalizations, focusing on why interventions work or not?

### Inclusion and Exclusion Criteria

Inclusion and exclusion criteria were specified using the PICo framework (Population, phenomena of Interest and Context) (24). Articles available in full text in English, Danish, Swedish, or Norwegian were considered eligible for inclusion if they met the specified criteria (Table 1). Studies that focused on people with mental illness, intellectual disabilities, palliative care needs, and pregnant women were excluded, since they might have special needs for secondary healthcare.

**Table 1:** Inclusion and exclusion criteria.

	**Inclusion criteria**	**Exclusion criteria**
Population	Citizens (age: ≥18 years) Healthcare professionals from the primary and secondary healthcare sectors, i.e., general practitioners; care managers, planners and coordinators; home nurses; community health workers; hospital doctors; hospital nurses; acute physicians	Non-adults (age: ≤ 17 years) People with mental illness People with intellectual disabilities Pregnant women People with palliative care needs Studies with other actors, e.g., family members (if results were entwined with results regarding the population eligible for inclusion in review)
Phenomena of interest	Interventions targeting the prevention of avoidable hospitalizations, and why interventions work or not Avoidable hospitalizations defined as hospitalizations that might have been prevented by effective intervention in primary care (11, 15)	Emergency admissions without reference to ACSCs Readmissions Studies focusing on whether patients were admitted to the appropriate hospital department or treatment
Context	Interventions performed in primary healthcare settings including home setting or community care setting Interventions across primary and secondary healthcare setting, e.g., intermediate care Studies undertaken in developed countries with universal healthcare, i.e. Austria, Belgium, Denmark, Finland, France, Germany, Greece, Ireland, Italy, Luxembourg, Netherlands, Portugal, Spain, Sweden, United Kingdom, Bulgaria, Croatia, Cyprus, Czech Republic, Estonia, Hungary, Latvia, Lithuania, Malta, Poland, Romania, Slovakia, Slovenia, Iceland, Norway, Switzerland, Australia, Canada, Japan, New Zealand (30, 31)	Interventions performed exclusively in a hospital setting
Study design	Quantitative, qualitative and mixed methods studies, including all study designs Mixed method studies were considered, if data from the quantitative or qualitative components could be clearly extracted	Reviews Conference abstracts Opinion letters Editorials Book chapters Protocols

### Search Strategy

A three-step search strategy (32) was used to identify potential relevant studies, which was developed and performed in collaboration with a specialist research librarian from Royal Danish Library, Faculty of Health, Aarhus University. There were no restrictions on the publication period. The systematic search was conducted in June 2019. First, an initial search in PubMed and Scopus was undertaken to identify relevant search terms. Reviews, articles, and policy documents on avoidable hospitalizations were identified (12–14, 23, 33). This was followed by an analysis of the words contained in titles, abstracts and index terms, which included, for example, “ambulatory care sensitive conditions” (13, 15) and “potentially preventable hospitalizations” (14). Relevant index terms were used to develop a full search strategy for PubMed (Supplementary Material 4). Second, a comprehensive search was undertaken in six databases: Scopus, PubMed, Cochrane Library, SveMed+, CINAHL and Embase. These databases were chosen because they cover the nursing, medical, and public health perspectives contained in the aim. The final search strategy included search terms related to: (1) avoidable admission (e.g., preventable admission, ambulatory care sensitive) and (2) the context; developed countries with universal healthcare (e.g., Austria, Belgium, Denmark, and Finland). This search strategy captured studies on avoidable hospitalizations undertaken in developed countries. The search strategy, including all identified keywords and index terms, was adapted for each database. Third, the reference lists of all studies meeting the inclusion and exclusion criteria were screened for additional studies. Following the search, all identified records were uploaded to Covidence, and duplicates were removed.

### Study Selection

Titles and abstracts were screened, and potentially relevant articles were read in full text, independently by CNL and MJJ, and compared to the inclusion and exclusion criteria. Any disagreements that arose between CNL and MJJ in the study selection process were resolved through discussion or involvement of a third reviewer (MB).

### Quality Assessment

Studies that did not meet the quality criteria (Table 1) were excluded to ensure that all findings included in the final syntheses were considered valid evidence useful to inform practice. The quality of qualitative studies and the qualitative component of mixed methods studies were assessed through the use of the JBI Critical Appraisal Checklist for Qualitative Research (34) by two reviewers independently (CNL and MB). The criteria focused on congruity and addressed aspects of validity in qualitative research reports. Articles meeting the five criteria focusing on dependability were included (35) [Critical Appraisal Checklist, questions (Q): Q2, Q3, Q4, Q6, Q7 (34)]. The quality of the quantitative studies were assessed through the use of the JBI critical appraisal checklists for randomized controlled trials, quasi-experimental studies, cohort studies, case-control studies, case-series and cross-sectional studies (36, 37). Each article was assessed by two reviewers independently (CNL and either MJJ or AHR). Prior to the assessment, the reviewers discussed the criteria of importance for risk of bias, and agreed that articles meeting the criteria regarding validity, reliability, and use of appropriate statistical methods should be included. Only articles complying with the established thresholds were included in the final review to strengthen the validity of the integrated findings, and any disagreements were resolved through discussion between the two reviewers, or with a third reviewer (MJJ or AHR).

### Data Extraction

Data were extracted in accordance with the JBI Mixed Methods Data Extraction Form following a Convergent Integrated Approach (24). First, the main characteristics of the studies, including country, aim, study design, population, phenomena of interest (intervention components), context, and results related to the aim, were extracted by CNL (38). Second, quantitative and qualitative data were extracted through the use of the review question. Quantitative data extracted comprised data-based outcomes and textual descriptions of the results, and qualitative data extracted comprised themes or subthemes with corresponding illustrations (a direct quotation from a citizen or healthcare professional, an observation or other supporting data from the article). If articles included findings from a literature review in the results section, or the perspectives of other stakeholders, e.g., relatives, only data representing the perspectives of the citizens and healthcare professionals were extracted. Meaning units were extracted through in-depth reading of articles' result sections and continuous discussion between CNL and MJJ, strengthening validity and reliability of extracted data. The data material is available upon request to CNL.

### Data Synthesis

The extracted data were analyzed using content analysis (26–29), as this is an open and systematic approach to identify and extract data of relevance for focused questions, assemble extracted data in descriptive categories, and produce integrated findings across categories. First, extracted quantitative data were transformed into textual descriptions, and these were combined with the extracted qualitative data. Second, all assembled data were categorized based on similarity in meaning, i.e., data addressing similar aspects of interventions targeting avoidable hospitalizations from the perspectives of the citizens and the healthcare professionals were compiled and labeled with concise descriptions. Third, categories were pooled based on similarity in meaning and were synthesized into integrated findings to produce a set of line of action statements. The categorization and synthetization were performed in continuous discussions between CNL, MJJ and MB to strengthen the validity and reliability of the extracted data, categories and integrated findings.

## Results

### Study Inclusion

**Figure 1:**
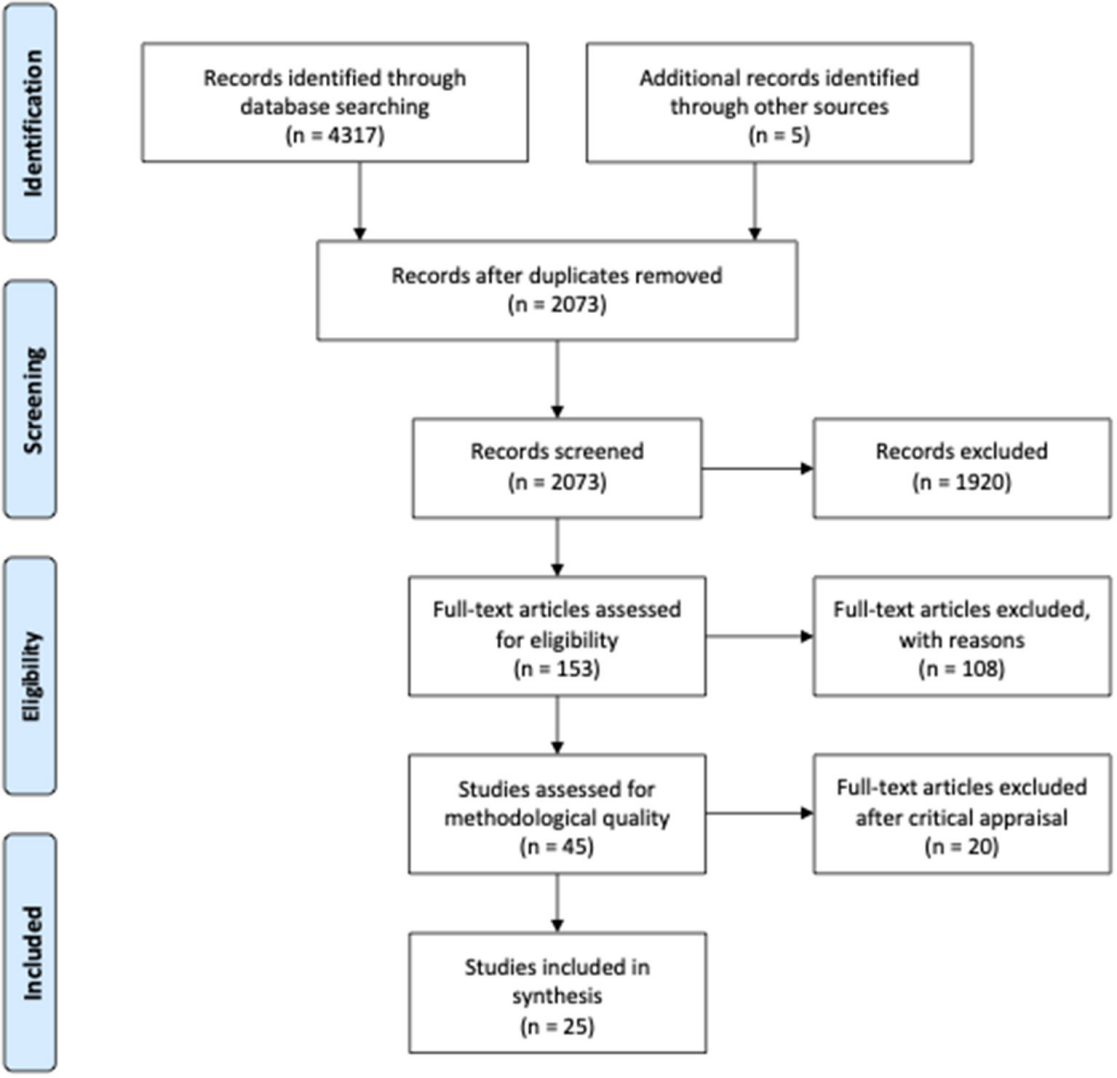
PRISMA flow diagram.

A total of 4317 articles were identified through the systematic search, and a hand search resulted in five additional articles. Citations were imported into Mendeley and Covidence, and 2,249 duplicates were removed. In Covidence, the 2073 citations were screened by reading title and abstract. The main reason for exclusion after screening of title and abstract was that articles did not meet the inclusion criteria regarding context, such as studies conducted in countries without universal healthcare, e.g., the United States. A total of 153 articles were read in full text, and 108 were excluded. The main reason for exclusion was that the articles did not provide evidence on intervention components that intend to prevent avoidable hospitalizations. In total, 45 articles (three mixed methods studies, nine qualitative studies, and 33 quantitative studies) matched the eligibility criteria, and these were critically appraised prior to inclusion [Figure 1, PRISMA Flow Diagram, adapted from Moher et al. (39)].

### Methodological Quality

A total of 25 articles (five qualitative studies and 20 quantitative studies) were considered to be of acceptable methodological quality and were included in the final review (quality appraisal results are stated in Supplementary Material 5). Of the 33 quantitative studies, 13 were excluded, as the studies did not respond to the criteria ensuring outcome assessment validity and/or because of insufficiency in the statistical analyses. For example, studies were not included, when lacking clear inclusion criteria, clarity in methods used for identification of the patient condition, and when lacking complete inclusion of participants. Of the nine qualitative studies, four were excluded, as these did not meet all five criteria, and thus congruity could not be ensured. Specifically lack of clarity regarding study participants and methodology used for data collection and analysis were assessed as critical, which therefore led to exclusion. This was also the case with the three mixed methods studies of which none met the pre-established thresholds for the qualitative component. As both the qualitative and the quantitative component in the mixed methods studies need to meet the set criteria, these studies were excluded.

### Description of Included Studies

The quantitative studies used different designs, including cross-sectional (40), observational (6, 41–43), non-randomized controlled trials (44–50), randomized controlled trial (51), survey (52), case study (53, 54), cohort (55, 56), and evaluations based on comparative or before-and-after study design (43, 57). Two of the studies was based on the same study population (43, 58). The qualitative studies used different methodologies, including a case study using individual and focus group interviews (59), a descriptive study using individual and focus group interviews analyzed with content analysis (60), a comparative case study using interviews and field notes (61), a realist evaluation using focus group and individual interviews, medical case notes, and literature review (62), and a study using individual interviews and a thematic analysis approach (63) (Table 2).

**Table 2:** Main characteristics of included studies.

**Article citation**	**Country**	**Aim**	**Study design**	**Population (number, characteristics)**	**Phenomena of interest (intervention components)**	**Context**	**Results stating the effectiveness and/or meaningfulness of intervention components**
Barker et al. (40)	England	To assess whether continuity of care with a general practitioner is associated with hospital admissions for ambulatory care sensitive conditions for older patients	Observational study (cross-sectional study)	*n* = 230,472 patients (age: 62–82 years) who experienced at least two contacts with a general practitioner between April 2011 and March 2013 for ACSCs [22 conditions adapted from Bardsley et al. (64)]	Continuity of care Intervention components: Longitudinal continuity of care through the usual provider of care index (the proportion of a patient's contacts that was with their most regularly seen doctor)	Primary healthcare setting (general practice)	Higher continuity of care was associated with fewer admissions for ASCSs There was greater evidence for an association among patients who were heavy users of primary care. Heavy users also experienced more admissions for ASCSs than other patients Billot et al. (6)	Australia	To assess the impact of a chronic disease management program on hospital utilization, with a focus on avoidable hospital admissions	Observational study (cohort study)	*n* = 41,303 individuals with ACSCs (diabetes, hypertension, chronic obstructive pulmonary disease, congestive heart failure, and coronary artery disease)	Chronic disease management program Intervention components: (1) care coordination across sectors (acute, ambulatory, and community care from both public and private sectors) and clinical specialties, facilitated by program care coordinators, and (2) health coaching, including management of lifestyle risk factors and medications and self-management	Across primary and secondary healthcare settings	Participation in the intervention was associated with an increase in avoidable admissions compared to matched controls but no difference in the rate of other types of hospitalization or death
Fiorentini et al. (41)	Italy	To assess the influence of different programs that ensure extra payments to general practitioners for containing avoidable hospitalizations	Observational study (case series)	*n* = 2,784,099 patients (age: 18–74); *n* = 3,095 general practitioners (with above 100 patients)	Incentives in primary care in relation to ACSCs [ACSCs as defined by Billings et al. (65)] Intervention components: (1) pay-for-performance; a link between financial transfer	Primary healthcare setting (general practice)	Pay-for-participation programs was not significant Both pay-for-performance and pay-for-compliance influence the probability of avoidable admissions Financial transfers aimed at improving the appropriateness of hospital referrals through additional financial transfers to
					and target achievements to be verified ex-post, (2) pay-for-participation; encouraging physician participation in the management of ACSCs, and (3) pay-for-compliance; financial transfers to general practitioners who take part in various activities that promote cooperation with professionals in charge of other levels of care		general practitioners are effective with the list of 27 Diagnosis Related Groups (DRGs) as reference, while they are not if one considers ACSCs
Freund et al. (44)	Germany	To determine whether protocol-based care management delivered by medical assistants improves care in patients at high risk for future hospitalization in primary care	Two-year cluster randomized clinical trial	*n* = 2,076 individuals with type 2 diabetes, chronic obstructive pulmonary disease, or chronic heart failure and a likelihood of hospitalization in the upper quartile of the population	Protocol-based care management (a paper-based assessment checklist to reveal individual needs and resources) Intervention components: (1) structured assessment, (2) action planning, and (3) monitoring delivered by medical assistants	Primary healthcare setting (general practice)	At 12 months, about 37% of the patients had been hospitalized at least once, and the number of all-cause hospitalizations per patient did not differ significantly between groups The number of chronic obstructive pulmonary disease-related hospitalizations was lower in the intervention group No significant differences in the number of diabetes-related or heart failure–related hospitalizations were found
Glasby et al. (59)	England	To explore the views of intermediate care leads on the benefits and challenges of implementing intermediate care policy, and 2) To assess the impact of intermediate care on the	Qualitative study using individual and focus group interviews	*n* = 82 healthcare professionals (key managers, and practitioners involved in the planning, management and delivery of intermediate care)	Intermediate care services Intervention components: (1) individual care plan, (2) a planned outcome of maximizing independent and typically enabling patients/users to resume living at	Primary healthcare setting, and across primary and secondary healthcare settings	Three main themes: (1) Intermediate care as part of a spectrum of services and as a positive alternative to hospital, (2) Difficulties in the relationship with acute care; issues for hospital staff, and (3) Difficulties in the relationship with acute care; issues for intermediate care staff
		service system as a whole and on individual users			home, (3) time-limited, normally no longer than six weeks and frequently as little as 1–2 weeks or less, and (4) involve cross-professional working, with a single assessment framework, single professional records and shared protocols		

Fourteen articles reported on interventions across primary and secondary healthcare settings, and 11 articles focused on interventions in primary healthcare settings. The interventions primarily targeted three groups: elderly with contact to the healthcare sector, people with chronic disorders, and healthcare professionals. The articles defined avoidable hospitalizations as hospitalizations that were objectively based on a primary diagnosis or a subjective evaluation by a general practitioner or nursing home staff. Articles using the term ACSCs commonly included diabetes, chronic obstructive pulmonary disease, and congestive heart failure (6, 41, 44, 46, 48, 51, 52). Two studies used indicators of avoidable hospitalizations relating to policy targets in the form of the diagnostic related groups (DRGs) (41) and measures of patient safety (48). Five studies focused on interventions targeted people with diabetes (42, 50, 55–57). One study presented an intervention targeting uncomplicated rectal bleeding (53). Two articles presented results from both healthcare professionals' and older people's perspectives (62, 63). One study included residents and their family members; from this study, only results related to the residents' perspective were extracted (60).

### Categories

From the 25 articles, 99 meaning units were extracted. From these, four categories were generated, describing intervention components that intend to prevent avoidable hospitalizations, focusing on why interventions work or not: (1) A trustful relation between the citizen and healthcare professional is a prerequisite for preventing hospitalizations, (2) Multidisciplinary and cross-sectoral teamwork is established to prevent avoidable hospitalizations, (3) Integration of preventive interventions in healthcare services prevent avoidable hospitalizations, and (4) Targeted tools guide and support primary healthcare professionals in initiating preventive interventions (Table 3).

**Table 3:** Summary of the main findings.

**Categories**	**Number of meaning units**	**Contributing articles**	**Integrated findings**	**Lines of Action**
(1) A trustful relation between the citizen and healthcare professional is a prerequisite for preventing hospitalizations	31	8 articles (40, 42, 52, 59–63)	Addressing individual needs through care continuity and coordination prevent avoidable hospitalizations	• Care practices should address both health-related issues and individual needs. • Clinical practice needs to be transformed to facilitate trustful relations between the citizen and healthcare professional, to allow healthcare professionals provide continuous and coordinated care, and to increase the involvement of the individual in preventive care practices. • Roles and responsibilities in multidisciplinary collaborations should be determined before initiation.
(2) Multidisciplinary and cross-sectoral teamwork is established to prevent avoidable hospitalizations	28	15 articles (6, 44, 45, 47–51, 56, 57, 59–63)		
(3) Integration of preventive interventions in healthcare services prevent avoidable hospitalizations	25	6 articles (41, 46, 55, 59, 61, 63)	Recognizing preventive care as an integrated part of the healthcare work to prevent avoidable hospitalizations	• Preventive care should be an integral part of the care work to ensure patient safety. • Healthcare administers and policymakers should support the preventive care by providing targeted educational material for healthcare professionals and simple web-based IT platforms for sharing information across healthcare settings. • Available tools need to take a broader perspective on individual health and functioning to prevent avoidable hospitalizations among those with complex care needs.
(4) Targeted tools guide and support primary healthcare professionals in initiating preventive interventions	15	5 articles (43, 46, 53, 56, 61)		

The four categories describe intervention components that intend to prevent avoidable hospitalizations, focusing on why interventions work or not, from the perspectives of citizens (age: ≥18 years) and healthcare professionals from the primary and secondary healthcare sectors.

#### A Trustful Relation

A trustful relation between the citizen and the healthcare professional enhanced the citizen's motivation and engagement in health-related preventive interventions, thus a trustful relation can be a prerequisite for preventing avoidable hospitalizations.

A trustful relation was established through specific actions, including solving practical issues, availability of care and responsiveness to the individual needs (59, 60, 62, 63). These actions gave rise to continuous contacts between the healthcare professional and the citizen, and thereby contributed to a trustful relation. Healthcare professionals providing home care can prevent avoidable hospitalizations, e.g., nurse practitioners in long-term care settings (60) and multidisciplinary teams providing home care to elderly citizens who prefer to stay in their own home and community (62). Trustful relations were built by providing *practical advice* and *solving important practical issues* that were not directly health-related (60, 62), such as installing loft insulation in the home (62). In addition, trustful relations were built through the *availability of care and responsiveness* to individual care needs, e.g., by spending extra time for the initial assessment, by providing more time for patient care, by having sufficient in-depth knowledge to deliver targeted information and emotional support, by ensuring continuity of care, and by emphasizing the personal relation (59, 60, 62, 63).

A thorough initial assessment enabled some patients to stay at home. It also provided them with an integrated care package, involving comprehensive care delivered by a team of nurse practitioners, specialist nurses, healthcare assistants, and care coordinators (62). More patient care helped older people regain their independence, which was seen as a key feature of intermediate care, involving individual care plan, a planned outcome, time-limited care, and cross-professional working (59, 63). Many patients favored the intermediate care services over hospital environments, appreciated the homely atmosphere and the locality of facilities, and felt that healthcare professionals had more time for patient care (63).

From the healthcare professionals' perspectives, having in-depth knowledge of the older people at long-term care homes and the ones living at home to meet their individual care needs was important to prevent avoidable hospitalizations (60, 62). From a nurse practitioner perspective, an important aspect of providing person-centered care was to notice subtle changes in health status, which required intimate knowledge of often complex health conditions, when descriptions focused primarily on health status (60). Then it was possible to meet the resident's need for informational and emotional support by keeping the resident (and family) informed about changes in health status or medication (60).

The *continuity of care* and the *personal relation* between older residents living at home and their healthcare professionals were important components in preventing avoidable hospitalizations. For example when patient's healthcare needs was pre-emptied the weekend (62); and through long-standing relationship between patient and general practitioner (40, 42, 52). Among people with diabetes, the optimal (maximum) time interval between general practitioner visits was 9–13 months for people with no diabetes complications, 5–11 months for people with 1–2 diabetes complications, and 4–9 months for people with 3+ diabetes complications if the visits were to protect against avoidable hospitalizations (42).

#### Multidisciplinary and Cross-Sectoral Teamwork

Multidisciplinary and cross-sectoral teamwork was established to meet complex care needs and to prevent avoidable hospitalizations, yet evidence on multidisciplinary care efforts was inconsistent. Nevertheless, good interdisciplinary collaboration between the healthcare professionals can enhance care coordination.

*Multidisciplinary teams* were established to overcome fragmentation and deliver better health outcomes for people with chronic diseases. However, there is mixed evidence on the effectiveness of multidisciplinary team-based practices, both across primary care institutions (45, 50, 57), and across primary and secondary care (6, 47–49, 56). A multidisciplinary team case management intervention, involving regular review and monitoring combined with an individual care plan for older people with ACSCs, had limited effect on reducing avoidable hospitalizations (48). Further, citizens with diabetes who received care managed by a physician outside of a primary care network were more likely to be hospitalized with ACSCs compared to citizens with diabetes who received care coordinated by a physician within the primary care network together with other healthcare professionals, but the differences between the two groups were small (56). Meanwhile, positive results were found in a multidisciplinary diabetes service on reducing avoidable hospitalizations when the multidisciplinary team comprised an endocrinologist, advanced-skilled general practitioners, a credentialed diabetes educator, and a podiatrist (50). Further, assigning a dedicated general practitioner to a nursing home was effective in preventing avoidable hospitalizations; this was explained by more efficient communication between the nurses at the nursing homes and the general practitioners (49).

From the healthcare professionals' perspectives, good interdisciplinary collaboration could enhance care coordination (60, 62), involving good working relationships between community-based healthcare teams and other services, such as outpatient clinics specializing in falls, general practitioner surgeries, and local councils enhanced care coordination (62).

Clear *roles* and clear *responsibilities* were important to provide *coordinated care* and to prevent avoidable hospitalizations when multiple healthcare professionals were involved and when services were constituted in home, municipality and hospital settings (59–63). For example, care plans that were left in the patient's home were difficult to keep up to date, which challenged the care coordination, when several healthcare professionals were involved (62). Confusion regarding the roles and responsibilities in multidisciplinary care work posed a risk for avoidable hospitalizations (59, 63). Consensus should be reached on the roles and responsibilities in the multidisciplinary teamwork, e.g., through discussions on when and where the care work needs to be done, and by whom (59). For example, team-based interventions outlining the healthcare professionals' preliminary roles and responsibilities in the care efforts were found to reduce avoidable hospitalizations (47, 51). In intermediate care facilities, where the care work was performed across settings such as hospital, general practice, nursing home, and residential home settings, a variety of prescribing arrangements in the distinct care settings resulted in confusion regarding prescribing responsibilities, thus posing a risk for patient safety and avoidable hospitalizations (63). To overcome confusion regarding roles and responsibilities in the provision of integrated care services across health disciplines and health institutions, it was beneficial and fostered coordination of care when a healthcare professional or administrative staff with a coordinating function had the overall responsibility for organizing the work (60, 61).

#### Integration of Preventive Interventions in Healthcare

A focus on treatment in the healthcare system dominated preventive practices. Consequently, preventive services were underused. Financial incentives to general practitioners were provided to promote interventions targeted avoidable hospitalization.

Preventive interventions were established in the *interface* between primary and secondary care settings, as *alternatives* to hospital-based care (e.g., intermediate care institutions and emergency beds) (46, 63). Increasing emergency bed capacity within the primary care sector was found to reduce avoidable hospitalizations related to asthma, angina, and chronic obstructive pulmonary disease (46). Further, intermediate care was introduced as a facility organizationally located between primary and secondary care, across which medicines have to be managed (59, 63). The care practices performed across primary and secondary care brought a shift in the responsibility for the care practices between the healthcare professionals, but also between the healthcare professionals and the citizen. In intermediate care settings, the patients expected the healthcare staff to take control of their medicine as being in a healthcare facility, while the healthcare professionals found that they needed to be in control of the patients' medicines to ensure patient safety (63). Self-management interventions targeted people with chronic disorders showed no effect on avoidable hospitalizations (6, 44). Rather, the increased focus on high-risk patients resulted in the diagnosis of additional conditions requiring hospitalization (6), and a longer intervention period was necessary to reveal positive results (44).

The *division between treatment and preventive care* entails difficulties in the implementation of interventions targeting the prevention of avoidable hospitalizations (59, 63). From the perspective of healthcare staff in intermediate care settings, the preventive services were underused as focus was on reducing acute care pressures, and little awareness and understanding of intermediate care was seen among hospital staff (59). A key barrier for using preventive services was difficulties with referring to intermediate care, which made practitioners revert to specialized healthcare services (59).

*Financial incentives*, such as payment and compensation schemes, supported the recruitment of general practitioners to participate in preventive interventions and draw their focus to specific conditions deemed avoidable (41, 55, 61). General practitioners perceived such incentives as a *recognition* that additional work needed to be done (61). Financial rewards for preventing avoidable hospitalizations were especially effective for conditions selected as attention points by the healthcare authorities (41). Citizens with diabetes listed at a general practice receiving a higher share of payments through compensation schemes were less likely to experience avoidable hospitalizations (55).

#### Targeted Tools Guide and Support Primary Healthcare Professionals

Tailored tools to guide and support healthcare professionals in primary care could prevent avoidable hospitalizations, as these tools motivated to initiate initiatives by drawing their attention to specific conditions deemed avoidable.

*Clinical decision-making tools* and *web-based IT platforms* were provided to general practitioners, which was found supportive to identify patients at high risk of avoidable hospitalizations, and thus facilitated the initiation of targeted preventive services (53, 54, 61). A fast-track consultation providing general practitioners with a direct phone line to hospital-based specialists played a role in the management of patients' treatment plans and in the prevention of avoidable hospitalizations (54). Also a triage tool that helped determine whether people with uncomplicated rectal bleeding needed to be hospitalized (53), and web-based tools providing focused and simple systems with direct access to relevant clinical information about residents (53, 54, 61) were found to prevent avoidable hospitalizations. General practitioners found that *intuitive systems*, which summarized relevant clinical information and allowed for recording of review decisions, were supportive in their clinical decision-making on whether and when to follow-up patients (61). General practitioners valued the *focused data* presentation and case-finding ability, as these features could help identify historical risk factors, which they were likely to overlook when using the usual medical record (61).

*Informational and educational material*, such as prescribing advice, structured written educational material, educational outreach visits, and tailored newsletters motivated healthcare professionals to engage in preventive interventions (61). Educational training and clinical decision support was shown to prevent avoidable hospitalizations in an intervention that included physician training and continuing education, physician participation in disease management programs, data-driven quality improvement, and computerized decision support (43, 58). General practitioners and primary healthcare professionals involved in preventive work and quality improvement, were motivated to engage in preventive interventions when the information material highlighted that the preventive care practices was about *patient safety* (61). These safety issues resonated with messages received from other sources; this provided high legitimation of the intervention and the implied actions, which motivated healthcare professionals facilitated to engage in an preventive intervention focusing on medicines prescribing in their clinical practices (61).

## Discussion

### Summary of Main Findings

This mixed methods systematic review aimed to identify and synthesize evidence on interventions targeting avoidable hospitalizations from the perspectives of the citizens and the healthcare professionals to improve the preventive healthcare services. The four categories were synthesized into two integrated findings. The first integrated finding “Addressing individual needs through care continuity and coordination prevent avoidable hospitalizations” underlines the importance of addressing individual needs to prevent avoidable hospitalizations through consistent, continuous and coordinated care across primary and secondary care settings. The second integrated finding state that “Recognizing preventive care as an integrated part of the healthcare work to prevent avoidable hospitalizations”. The distinction between prevention and treatment flows from the sectoral division between primary and secondary care, and this frames the healthcare professionals' opportunities and motivation to engage in preventive practices to reduce avoidable hospitalizations. An assessment of the certainty of the evidence was not conducted. Recommendations based on quantitative evidence are often assessed using the GRADE approach (32), and recommendations based on qualitative evidence are often assessed using either the GRADE-CERQual (66) or the ConQual approach (32). However, because of the complexities associated with recommendations based on both quantitative and qualitative evidence, it is currently not recommended to assess the certainty of such recommendations (24). Meanwhile, to ensure methodological rigor and scientific quality, the included studies all met the established threshold criteria in the quality assessment, thus the integrated findings are considered valid evidence to inform practice.

#### Integrated Finding 1: Addressing Individual Needs Through Care Continuity and Coordination Prevent Avoidable Hospitalizations

This integrated finding was generated from two categories: category (1) a trustful relation between the citizen and healthcare professionals is a prerequisite for preventing hospitalizations, and category (2) multidisciplinary and cross-sectoral teamwork is established to prevent avoidable hospitalizations. This synthesis emphasizes the importance of continuity of care, including a trustful relation between citizen and healthcare professional, as a driver of care coordination, and thereby providing interventions that adapts to individual needs to prevent avoidable hospitalization. A history of interaction with the same general practitioner was shown to prevent avoidable hospitalizations (40, 42, 52), which highlights the potential of structured and person-centered care plans conforming to individual needs (including both health-related and practical issues) to prevent avoidable hospitalizations (59, 60, 62, 63). Other studies have shown that the risk of hospitalization is affected by *disorder-related factors*, including progression of disease, multimorbidity, mental-physical comorbidity (67–70), by *person-related factors*, including delayed health seeking, lack of knowledge, awareness and understanding of condition, perceived stress, low income, education and self-care abilities (67, 71–75), and by *system-related factors*, including service capacity, lack of care continuity, and little coordination between healthcare sites (75, 76). This is in line with the WHO framework on integrated people-centered health services (17), which conceptualizes care continuity as a complex concept of four domains: (1) interpersonal, (2) longitudinal, (3) management, and (4) informational continuity. The WHO framework recognizes overlaps between care continuity and coordination, where continuity enables effective care coordination that adapts to individual needs to ensure that the care is integrated and person-centered across various care settings. Likewise, our review findings showed that assigning a dedicated general practitioner in a nursing home was effective in reducing preventable hospitalizations, and this was explained by more efficient communication between nurses at the nursing homes and general practitioners (49). Thus, preventive interventions should not only address the direct health-related issues; such interventions should also consider the citizen and the context, including this individual's living conditions and needs for support, to prevent avoidable hospitalizations.

This systematic review excluded studies on palliative care interventions and findings representing the perspectives of relatives. This was done to focus explicitly on citizens at risk of avoidable hospitalizations and the healthcare professionals involved in interventions aiming to prevent avoidable hospitalizations. Supplementing our review findings, another mixed methods systematic review (77) focused on palliative care patients and their families. This study reports on the experiences of patients and their families, highlighting their needs of developing continuous relationships with clinicians (relational continuity) and coordinated, comprehensive information sharing within a range of services or professionals (informational continuity). The review stated a reduction in avoidable hospital admissions owing to interventions designed to promote informational and relational continuity (77). Further, a lack of informational and relational continuity negatively impacted the experiences of people with palliative care needs and their families, who had to act as a coordinator between the person with care needs and the healthcare professionals (77). Another study showed that older people valued good communication and a confidential and caring relationship with their primary care provider, as it facilitated their involvement in own care (78).

The present review findings are based on interventions targeting different groups, e.g., older people and people with chronic disorders, with seemingly various care needs; older people might have more complex needs as they are likely to be more cognitively or physically impaired because of their age, whereas people with diabetes without complications might have more direct needs. Yet, despite these potential differences in care needs, the review highlights the importance of care continuity, including a trustful relation between the citizen and healthcare professional. Accordingly, a study showed that consulting a primary care provider who was known and trusted was important to 62.6% of the respondents (79). Thus, care continuity can be underscored as an important component to prevent avoidable hospitalizations. This is also supported by the practice priorities put forward in the WHO framework (17), which aims to guide practitioners, providers, organizations, and system leaders to organize, manage, and deliver care that best meets citizen's health needs.

Further, this synthesis of evidence emphasizes the importance of clarifying the roles and responsibilities in team-based interventions to foster continuity and coordination of care. The results revealed underlying misunderstandings of other healthcare professionals' roles, and confusion regarding the division of responsibilities in multidisciplinary efforts, which may pose a patient safety risk. Team-based interventions fostered continuity and coordination of care when a healthcare professional or administrative staff with a coordinating function had the overall responsibility for organizing the work (60, 61). Another study showed that healthcare professionals were less motivated to participate in integrated care when the specific roles and responsibilities were unclear in the interdisciplinary team, implying a need for shared responsibility to engage all healthcare professionals (80). To build coordinated care practices, the roles and responsibilities should be outlined prior to initiation of the collaboration, for example through discussions of when and where the care work should be done, and by whom.

Fifteen of the 25 included articles provided evidence on multidisciplinary and cross-sectoral teamwork to prevent hospitalization (7, 45, 46, 48–52, 57, 58, 60–63, 67). The studies revealed inconsistent results for the prevention of avoidable hospitalizations, with fewer avoidable hospitalizations related to diabetes and limited effect among older people with ACSCs. A possible explanation could be that different types of coordination and collaboration between healthcare professionals is needed, depending on whether the person have complex care needs requiring involvement of different healthcare specialists, or more simple care needs (e.g., uncomplicated diabetes) for which healthcare and actions have been further developed and targeted (81). Nevertheless, the fragmentation of care practices, i.e., provision of care in various settings, combined with the involvement of different healthcare professionals remain barriers for ensuring continuous care and coordinated care practices.

#### Integrated Finding 2: Recognizing Preventive Care as an Integrated Part of the Healthcare Work to Prevent Avoidable Hospitalizations

The second integrated finding was generated from category 3) Integration of preventive interventions in healthcare services prevent avoidable hospitalizations, and category 4) Targeted tools guide and support primary healthcare professionals in initiating preventive interventions. This synthesis reveals that the sectoral divisions between primary and secondary care manifest in a demarcation between prevention and treatment, which influences the care practices aiming to prevent avoidable hospitalizations. To motivate and engage healthcare professionals in preventive interventions, financial incentives and supportive tools are provided to help stimulate especially primary care professionals in addressing avoidable hospitalizations. Correspondingly, the WHO framework (17) highlights financial incentives, technology, and education as enablers of care coordination that enhance the management and the informational continuity of care. The WHO framework recognizes that financial incentives aligned with shared outcomes of specific care practices are important components to provide more comprehensive care along the entire care pathway (17). In addition, review findings have contributed with evidence on the effects of financial incentives (41, 55, 61), clinical decision-making tools for general practitioners, and support systems for clinical recording and patient reviews, which were all shown to prevent avoidable hospitalizations (53, 54, 61). Thus, to successfully engage healthcare professionals in preventive care efforts, intervention components should comprise financial incentives, branding of interventions, and information and educational material. These components have been shown to enhance the healthcare professionals' engagement, to bring their professional knowledge and values into play (e.g., increased patient safety or implied preventive actions), and to make the intervention ethically legitimate from a professional point of view. One excluded study, which did not meet the inclusion criteria as it evaluated the cost-effectiveness of a primary care program aimed at preventing avoidable hospitalizations (82). In this program, general practitioners were invited to manage patients while using any resources required up to a cost of approximately $266 per patient; the results showed that 104 patients of the 707 enrolled patients were admitted to hospital (82). Such primary care programs are supported by the WHO, which highlights the primary care sector as a central player in coordinating health services across various disciplines and organizational boundaries (7, 17). Based on our results, and in line with other studies (7, 83, 84), it can be concluded that a fragmented healthcare system tends to result in suboptimal care and poor quality of care, which may impose a risk for patient safety and avoidable hospitalizations. Consequently, prevention should form an integral part of the care provided to truly engage and motivate healthcare professionals in preventive care. Moreover, the preventive efforts should account for the citizens' individual needs, and this should be reflected in relevant educational material, in financial incentives, and at platforms for sharing information across healthcare settings.

In this review, studies on readmissions were excluded to correspond to the distinction between hospital admissions and readmissions applied in existing research articles on avoidable hospitalizations, but this operationalization might not be meaningful in practice as admissions and readmissions occur on a continuum. Supplementing our review results, a systematic review on readmissions highlighted the important role of hospitals in transitional care interventions and the coordination of chronic care to ensure better outcomes for patients and fewer readmissions (85). The review stated that most problems related to continuity of care occurred at transition points involving a lack of cross-boundary continuity between sites or providers, or a lack of flexibility in the coordination when there were major changes in the patients' needs (85). In line with this, our review results underscore the importance of flexible care plans and care coordination between healthcare professionals, especially for people with complex care needs. Additionally, the review contributes with evidence on intervention components that intend to prevent avoidable hospitalizations, and important findings on the challenges related to establishing care continuity and coordination. Further, the findings highlight the importance of addressing motivators and enablers to engage healthcare professionals and people with care needs in preventive interventions.

### Strengths and Limitations

To the best of our knowledge, this mixed methods systematic review offers the first synthesized combined qualitative and quantitative evidence on interventions targeting avoidable hospitalizations, focusing on why interventions work or not, from the perspectives of the citizens and the healthcare professionals. The citizen perspective contributed with knowledge on perceived care needs in relation to primary healthcare services. The healthcare professionals' perspectives contributed with knowledge on providing and engaging in preventive interventions.

The review has several important strengths. The broad operationalization of avoidable hospitalizations accounted for various terms and definitions used in the literature, which included a broad diversity of diseases rather than only few specific conditions. In accordance with the broad operationalization of avoidable hospitalizations, keywords were identified and included in the search strategy, e.g., avoidable admissions, ambulatory care sensitive conditions, preventable hospitalizations. However, studies using other terms than the ones included in our search were not identified, although these might have contributed with useful findings. Nevertheless, the broad operationalization of avoidable hospitalization ensured that articles on interventions targeting avoidable hospitalizations were identified. Moreover, the heterogeneity of the population strengthens the generalizability of the findings. Additional strengths were the external validity of the review and the inclusion of studies conducted in nine different countries with universal healthcare, representing different healthcare models, including national health service (Australia, Northern Ireland, England, Norway, Denmark, Italy), national health insurance (Canada), and social health insurance (France, Germany) (86, 87). Further, the systematic search strategy was developed to include not only English articles, but also articles published in Swedish, Norwegian and Danish. The qualitative content analysis helped synthesize different types of evidence, as it allowed us to transform complex meaning units into descriptive integrated findings to produce a set of line of action statements (88). The review holds some limitations stemming from the complexities associated with deriving recommendations from both qualitative and quantitative evidence, as there is currently no tool for assessing the certainty of the evidence of integrated findings (24). Yet, to compensate for the lack of quality assessment tools for the integrated findings, all articles were critically appraised, by two reviewers independently, prior to inclusion in the final review; this was done to strengthen the certainty of the recommendations for clinical practice (24).

In this review, none of the identified mixed methods studies corresponded to the criteria for acceptable methodological quality, and were thus excluded. Further, the transferability of the integrated findings was limited, as the interventions targeted primarily older people or people with a single chronic disorder (e.g., diabetes), and the included articles used different definitions of avoidable hospitalizations and follow-up periods. Some of the included articles reported an increase in avoidable hospitalizations, and some showed little effect of interventions, which suggests a need for longer interventions to reveal positive effects (6, 44, 48, 56). Nevertheless, the synthesized evidence presented in this review may be a valuable resource in the development and implementation of interventions aimed at reducing avoidable hospitalizations while accounting for both citizens' and healthcare professionals' perspectives. The review may also help ensure patient safety and improve the service quality in preventive healthcare.

### Implications for Practice

To inform practice, the two integrated findings were supplemented with lines of action statements (see Table 3 and Supplementary Material 3).

The first integrated finding (addressing individual needs) suggests that more attention should be given to the care practices that address both health-related issues and individual needs, as this combination seems to prevent avoidable hospitalizations. Therefore, clinical practice needs to be transformed to facilitate trustful relations between healthcare professionals and citizens, to allow healthcare professionals provide continuous and coordinated care, and to increase the involvement of citizens in preventive care practices. To strengthen the implementation of team-based interventions and to support continuity and coordination of care, the roles and responsibilities in multidisciplinary collaborations should be determined before initiation, for example who, when and where the care work should be done.

The second integrated finding (recognizing preventive care as an integrated part of the healthcare work) shows that, to engage and motivate healthcare professionals in preventive care that considers individual needs, preventive care should be an integral part of the care work to ensure patient safety. Healthcare administers and policymakers should support the preventive care by providing targeted educational material for healthcare professionals and simple web-based IT platforms for sharing information across healthcare settings. Financial incentives tend to draw the healthcare professionals' attention to specific conditions in the efforts to prevent avoidable hospitalizations. Yet, to prevent avoidable hospitalizations among those with complex care needs (e.g., older people living at home or nursing homes), the available tools need to take a broader perspective on individual health and functioning, as this would allow healthcare professionals to consider both health-related issues and mental/social conditions (e.g., living conditions).

### Implications for Research

This review reported limited availability of evidence on intervention components that intend to prevent avoidable hospitalizations, especially evaluations of interventions and qualitative studies from a citizen perspective. Future studies should aim to understand and explain why (or why not) interventions work to enhance the transferability of effective interventions to prevent avoidable hospitalizations. Additionally, there is a need for knowledge to clarify the important elements in social and informational support, and the drivers experienced by citizens in managing own health should be explored to facilitate active participation in preventive actions. Qualitative studies are needed to explore the perspectives of key actors involved in preventive healthcare interventions (e.g., healthcare managers, healthcare professionals, people with care needs and relatives), as such studies could provide evidence to guide the implementation of interventions. Systematic reviews investigating the perspectives of multiple stakeholders could provide new knowledge on specific intervention components fostering care continuity and coordination, which could lead to further development of evidence-based clinical guidelines on integrated care.

## Conclusions

This mixed methods systematic review presents the best available evidence on the interventions targeting avoidable hospitalizations. It provides a wider understanding of the practical applicability of interventions and the adherence to interventions. The review reports that a trustful relation between the citizen and healthcare professional is an important element of continuity of care to prevent avoidable hospitalizations. The continuous contact between the citizen and a trusted healthcare professional was a driver for care coordination. A healthcare professional with a coordinating function can involve other healthcare professionals in preventive efforts and give citizens access to relevant services. Preventive healthcare should be organized to facilitate the engagement of multidisciplinary healthcare professionals from across sectors to accommodate individual needs and to prevent avoidable hospitalizations. This approach takes a broader perspective to the traditional one-track focus on treatment in the healthcare system. The review results could serve as a valuable resource in the development and implementation of interventions to prevent avoidable hospitalizations, and may serve to improve patient safety and quality in preventive healthcare services.

## Data Availability Statement

The original contributions presented in the study are included in the article/Supplementary Material, further inquiries can be directed to the corresponding author.

## Author Contributions

The review protocol and design were framed and data analysis and syntheses were performed in continuous discussions between by CNL, MB, and MJJ. The systematic search was performed and the first draft of the manuscript text was written by CNL. Data was collected by CNL and MJJ. Quality appraisal was performed by CNL, MB, AHR, and MJJ. The manuscript was continuously reviewed by MJJ. All authors reviewed and approved the final manuscript.

## Funding

This project was supported by Innovation Fund Denmark, grant no. 7076-00015B. The funder had no role in the study design, data collection or analysis, decision to publish, or preparation of the manuscript.

## Conflict of Interest

AHR was employed by Enversion A/S. The remaining authors declare that the research was conducted in the absence of any commercial or financial relationships that could be construed as a potential conflict of interest.

## Publisher's Note

All claims expressed in this article are solely those of the authors and do not necessarily represent those of their affiliated organizations, or those of the publisher, the editors and the reviewers. Any product that may be evaluated in this article, or claim that may be made by its manufacturer, is not guaranteed or endorsed by the publisher.

## Abbreviations

ACSCs: ambulatory care sensitive conditions
OECD: Organization for Economic Co-operation and Development
WHO: World Health Organization.
