# Supplementary material for: Interventions to Prevent Potentially Avoidable Hospitalizations: A Mixed Methods Systematic Review
Source: Front Public Health. 2022 Jul 11;10:898359. doi: 10.3389/fpubh.2022.898359 (PMC9309492; doi:10.3389/fpubh.2022.898359)
Supplement: Supplementary file 1 [file Data_Sheet_1.pdf]

Preventing potentially avoidable admissions - a mixed methods systematic review protocol

Cecilie Nørby Thisted, Merete Bjerrum, Marianne Johansson Jørgensen

Citation

Cecilie Nørby Thisted, Merete Bjerrum, Marianne Johansson Jørgensen. Preventing potentially avoidable admissions - a mixed methods systematic review protocol. PROSPERO 2020 CRD42020134652 Available from:
https://www.crd.york.ac.uk/prospero/display_record.php?ID=CRD42020134652

Review question

Which intervention components are effective and/or meaningful in preventing potentially avoidable admissions from the perspectives of adults 18 years and above with ambulatory care sensitive conditions (ACSCs) and health professionals?

Searches

The systematic review is conducted in accordance with The Joanna Briggs Institute (JBI) framework for mixed methods systematic review, and the search strategy will be designed in accordance with the three-phase process as described in the Joanna Briggs Institute Reviewer's Manual (2017). The search strategy will be developed in collaboration with a specialist research librarian from Aarhus University Library, Health Sciences. The systematic search includes searching scientific databases for studies that match the pre-defined inclusion and exclusion criteria. Both qualitative and quantitative evidence will be reviewed, with publications available in full text with an English abstract considered for potential inclusion.

Search terms are identified corresponding to the PICO/PICo criteria. Key search terms include: avoidable admission OR preventable admission OR unnecessary admission OR inappropriate admission OR ambulatory care sensitive conditions OR ambulatory sensitive hospitalisation OR inappropriate hospitalisation OR unnecessary hospitalisation OR preventable hospitalisation OR avoidable hospitalisation.

The following scientific databases will be searched for relevant articles:

• Scopus

• Embase

• PubMed

• CINAHL

• The Cochrane Library

The reference lists of the included studies will be hand searched for additional relevant articles.

There will be no restrictions on the publication period. The search will be conducted in June-July 2019.

Reference: Aromataris E, Munn Z (Editors). Joanna Briggs Institute Reviewer's Manual. The Joanna Briggs Institute, 2017. Available from https://reviewersmanual.joannabriggs.org/

Types of study to be included

Inclusion: Quantitative and qualitative primary studies including all study designs. Mixed method studies were considered if data from the quantitative or qualitative components could be clearly extracted.

Exclusion: All types of reviews, conference abstracts and protocols.

Condition or domain being studied

The systematic review focus on effective and/or meaningful interventions targeting the prevention of

potentially avoidable admissions of adults 18 years and above with ACSCs.

A preliminary search in PubMed, PROSPERO and Cochrane Library revealed that a mixed methods systematic review on effective and/or meaningful interventions targeting the prevention of potentially avoidable admissions has not yet been conducted. Literature reviews on similar thematic topics were identified. Purdy et al. (2013) focus on the care of older people in summarising literature predicting and preventing avoidable hospital admissions, Thomas et al. (2014) focus on pharmacist-led interventions to reduce unplanned admissions for older people and Shepperd et al. (2016) focus on the effectiveness and cost of managing older people with admission avoidance hospital at home. Meanwhile, these reviews are unsystematic, narrowed to older people and/or focus on measures of effectiveness and costs.

References:

Purdy S & Huntley A. Predicting and preventing avoidable hospital admissions: a review (2013)

Thomas et al. Pharmacist-led interventions to reduce unplanned admissions for older people: a systematic review and meta-analysis of randomized controlled trials. Age and Aging 2014; 43: 174-187

Shepperd S et al. Admission avoidance hospital at home. Cochrane Database of Systematic Reviews 2016, Issue 9. Art. No.: CD007491. DOI: 10.1002/14651858.CD007491.pub2.

Participants/population

Inclusion: Males and females 18 years and above with ACSCs, and healthcare professionals from the primary and secondary healthcare sector, e.g., general practitioners, physicians, home nurses, emergency nurses, community health workers, physiotherapists, dietitians, occupational therapists, hospital doctors, hospital nurses, acute physicians

Exclusion: Non-adults (17 years or younger), people with mental illness, people with intellectual disabilities and pregnant women. Studies focusing on the perspectives of other stakeholders including policy-makers and relatives.

Intervention(s), exposure(s)

Inclusion: Healthcare interventions targeting the prevention of potentially avoidable admissions. The focus is on healthcare interventions performed in primary or secondary healthcare settings or across primary and secondary healthcare settings.

Exclusion: Interventions in psychiatric hospitals and mental health units.

Comparator(s)/control

Not applicable.

Context

Inclusion: Studies undertaken in developed countries with universal health care, i.e. Austria, Belgium, Denmark, Finland, France, Germany, Greece, Ireland, Italy, Luxembourg, Netherlands, Portugal, Spain, Sweden, United Kingdom, Bulgaria, Croatia, Cyprus, Czech Republic, Estonia, Hungary, Latvia, Lithuania, Malta, Poland, Romania, Slovakia, Slovenia, Iceland, Norway, Switzerland, Australia, Canada, Japan, New Zealand

References:

McKee, M., Balabanova, D., Basu, S., Ricciardi, W., & Stuckler, D.

(2013). Universal health coverage: A quest for all countries but under

threat in some. Value in Health, 16, S39–S45.

https://doi.org/10.1016/j.jval.2012.10.001

United Nations. (2018). World Economic Situation and Prospect 2018

report. New York: United Nations.

Main outcome(s)

In accordance with the JBI methodology for mixed methods systematic reviews (2017), outcome is not used as a criterion for inclusion. Studies that contains knowledge on the effectiveness and/or meaningfulness of intervention components that target avoidable admissions are considered for inclusion in the review.

Effectiveness refers to the extent to which an intervention achieves the intended result or outcome with regard to preventing potentially avoidable admissions (Jordan et al., 2019)

Meaningfulness refers to how an intervention or activity is experienced by and individual or group and the meanings they ascribe to that experience (Jordan et al., 2019)

Reference:

Jordan, Z., Lockwood, C., Munn, Z., & Aromataris, E. (2019). The

updated Joanna Briggs Institute Model of Evidence-Based

Healthcare. International Journal of Evidence-Based Healthcare,

17(1), 58–71. https://doi.org/10.1097/XEB.0000000000000155

Lizarondo L, Stern C, Carrier J, Godfrey C, Rieger K, Salmond S, Apostolo J, Kirkpatrick P, Loveday H. Chapter 8: Mixed methods systematic reviews. In: Aromataris E, Munn Z (Editors). Joanna Briggs Institute Reviewer's Manual. The Joanna Briggs Institute, 2017. Available from https://reviewersmanual.joannabriggs.org/

* Measures of effect

None.

Additional outcome(s)

None.

* Measures of effect

None.

Data extraction (selection and coding)

Following the search, all identified citations will be loaded into Mendeley and Covidence, and duplicates removed.

Titles and abstracts will then be screened by CNT for assessment against the inclusion criteria for the review. The full text of selected citations will be assessed in detail against the inclusion criteria by two independent reviewers (CNT and MJJ).

Any disagreements that arise between CNT and MJJ at each stage of the study selection process will be resolved through discussion, or with a third reviewer (MB).

Data extraction:

Since the decisions regarding inclusion of relevant articles are based on the quality assessment, extraction of studies' main characteristics, and extraction of meaning units answering the review question will be

performed after the quality assessment of articles that match the predefined inclusion criteria.

Risk of bias (quality) assessment

Articles that match the predefined inclusion and exclusion criteria will be critically appraised prior to inclusion in the final review. Quantitative articles will be assessed using the standardised appraisal tools developed by the JBI. Qualitative articles will be assessed using the standardised appraisal checklist from the JBI Qualitative Assessment and Review Instrument. Two reviewers will independently appraise the articles and discuss the appraisals to reach consensus regarding inclusion or exclusion based on the methodological quality.

Reference: Aromataris E, Munn Z (Editors). Joanna Briggs Institute Reviewer's Manual. The Joanna Briggs Institute, 2017. Available from https://reviewersmanual.joannabriggs.org/

Strategy for data synthesis

The qualitative and quantitative evidence will be combined through a convergent integrated approach following the JBI methodology for mixed methods systematic reviews (2017), as this method allows the categorisation of different types of evidence based on similarity in meaning to produce a set of integrated findings.

Quantitative and qualitative data will be extracted from studies included in the review by two independent reviewers (CNT and MJJ) using Covidence. The data extracted will include specific details about the populations, study methods, phenomena of interest, context and outcomes of relevance to the review question. Specifically, quantitative data will comprise of data-based outcomes of descriptive and/or inferential statistical tests. In addition, qualitative data will comprise of themes or subthemes with corresponding illustrations, and will be assigned a level of credibility.

The quantitative data will be transformed into textual descriptions or narrative interpretation of the quantitative results so as to respond directly to the review question. Following the integrative approach, the qualitised data will then be assembled with the qualitative data. Assembled data are categorised and pooled together based on similarity in meaning to produce a set of integrated findings in the form of categories.

Any disagreements that arise between CNT and MJJ in the data extraction process will be resolved through discussion, or by consulting a third reviewer (MB).

Reference:

Lizarondo L, Stern C, Carrier J, Godfrey C, Rieger K, Salmond S, Apostolo J, Kirkpatrick P, Loveday H. Chapter 8: Mixed methods systematic reviews. In: Aromataris E, Munn Z (Editors). Joanna Briggs Institute Reviewer's Manual. The Joanna Briggs Institute, 2017. Available from https://reviewersmanual.joannabriggs.org/

Analysis of subgroups or subsets

None planned.

Contact details for further information

Cecilie Nørby Thisted
cnth@ph.au.dk

Organisational affiliation of the review

Aarhus University, Department of Public Health
http://health.au.dk/en/

Review team members and their organisational affiliations

Dr Cecilie Nørby Thisted. Aarhus University, Department of Public Health
Assistant/Associate Professor Merete Bjerrum. Aarhus University, Department of Public Health
Dr Marianne Johansson Jørgensen. Regional Hospital in Horsens, Department of Research

Type and method of review
Systematic review, Other

Anticipated or actual start date
01 May 2019

Anticipated completion date
31 December 2019

Funding sources/sponsors
Funding provided by the Grand Solution Program, the Innovation Fund Denmark.

Conflicts of interest

Language
English

Country
Denmark

Stage of review
Review Ongoing

Subject index terms status
Subject indexing assigned by CRD

Subject index terms
Hospitalization; Humans; Patient Admission

Date of registration in PROSPERO
03 February 2020

Date of publication of this version
03 February 2020

Details of any existing review of the same topic by the same authors

Stage of review at time of this submission

**Stage**	**Started**	**Completed**
Preliminary searches	Yes	No
Piloting of the study selection process	No	No
Formal screening of search results against eligibility criteria	No	No
Data extraction	No	No
Risk of bias (quality) assessment	No	No
Data analysis	No	No

The record owner confirms that the information they have supplied for this submission is accurate and complete and they understand that deliberate provision of inaccurate information or omission of data may be construed as scientific misconduct.

The record owner confirms that they will update the status of the review when it is completed and will add publication details in due course.

Versions

03 February 2020

PROSPERO
This information has been provided by the named contact for this review. CRD has accepted this information in good faith and registered the review in PROSPERO. The registrant confirms that the information supplied for this submission is accurate and complete. CRD bears no responsibility or liability for the content of this registration record, any associated files or external websites.
