# Supplementary material for: Interventions to Prevent Potentially Avoidable Hospitalizations: A Mixed Methods Systematic Review
Source: Front Public Health. 2022 Jul 11;10:898359. doi: 10.3389/fpubh.2022.898359 (PMC9309492; doi:10.3389/fpubh.2022.898359)
Supplement: Supplementary file 2 [file Data_Sheet_2.docx]

**Additional File 2.** JBI Guidelines for Reporting.

The mixed methods systematic review was conducted following the JBI convergent integrated approach, therefore the JBI Guidelines for conducting and reporting a mixed methods systematic review is the appropriate guideline for reporting

<https://jbi-global-wiki.refined.site/space/MANUAL/3318284366/8.5.1+++Mixed+methods+systematic+review+using+a+CONVERGENT+INTEGRATED+approach+to+synthesis+and+integration>

The table include the JBI reporting items accompanied by the heading from the manuscript, and if relevant, sub-headings and Table/Figure/Additional File reference, stating the manuscript section that report how the single items were accomplished.

| **Section/topic** | **#** | **Item**  **(a summary of the components in the JBI Manual for Conducting and Reporting a MMSR using a Convergent Integrated Approach)** | **Reported under**  **Heading #; Subheading ##** |
| --- | --- | --- | --- |
| **Title of a mixed methods systematic review** | | | |
| Title | 1 | The title should be informative and give clear indication of the topic of the MMSR. The title should always include the phrase “…: a mixed methods systematic review” to allow easy identification of the type of document it represents | Title page |
| **Abstract** | | | |
| Abstract | 2 | This section is a summary of the review in 500 words, stating the objective, methods, main findings and principal conclusions of the review. The abstract should not contain abbreviations or references. | Abstract |
| **Introduction** | | | |
| Rationale | 3 | Describe and situate the topic of interest under review. Definitions can assist to provide clarity. Where complex or multifaceted phenomena are being described, it may be important to detail the whole of the phenomenon for an international readership. Explanation of how the review question can be answered by both quantitative and qualitative studies is required as is an explanation on how the review will add to the evidence base or inform clinical practice.  A statement that a preliminary search of databases (with databases listed) has been undertaken and no existing or ongoing mixed method or systematic reviews on the topic have been identified should be provided. If other systematic reviews on the topic exist, indication on how the proposed systematic review differed should be detailed. | Introduction |
| Objective | 4 | Conclude with an overarching review objective that captures and aligns with the core elements of the inclusion criteria. | Introduction |
| **Review questions and Inclusion criteria** | | | |
| Review questions | 5 | The review question(s) should be explicitly stated in unambiguous terms | Methods; Design |
| Inclusion criteria | 6 | Inclusion criteria should be reasonable, sound and justified and address the elements in the PICo question(s). | Methods; Inclusion and Exclusion Criteria, Table 1 |
| **Methods** | | | |
| Methods | 7 | This section of the review is reserved for the methods used to conduct the review and should be presented under the relevant subheadings, including any deviations from the method outlined in the a priori protocol and a rationale. | Methods; Design |
| Search strategy | 8 | Provide explicit and clear information regarding all information sources that were used in the review, and the actual strategies used for searching. | Methods; Search Strategy |
| Study selection | 9 | Describe the actual process of study screening for all stages of selection (e.g. title and abstract examination; full text examination) and the actual procedures used for solving disagreements between reviewers. | Methods; Study Selection |
| Assessment of methodological quality | 10 | Specify the critical appraisal process and instruments that were used in the review process and the procedures for solving disagreements between reviewers. The details of the decision processes and criteria used for exclusion of studies based on the results of critical appraisal should be explicitly provided. All details about the scoring systems and the cut-off scores (if applicable) for inclusion of studies in the review should be described and justified.  Indicate what constituted acceptable levels of information for a study to receive a positive, negative or unclear response to a critical appraisal question and if applicable, the rationale and criteria for excluding studies on the basis of methodological quality. | Methods; Quality Assessment |
| Data extraction | 11 | Specify the data extraction process and instruments that were used in the review process and the procedures for solving disagreements between reviewers. | Methods; Data Extraction |
| Data transformation | 12 | Specify the data transformation process that was used to convert the extracted quantitative data into qualitized data to facilitate integration with data extracted from qualitative studies. | Methods; Data Extraction |
| Data synthesis and integration | 13 | Indicate that a convergent integrated approach was applied. The review should detail how the reviewers analyzed and integrated the data extracted from included quantitative, qualitative and mixed methods studies and detail the aggregative approach to integration. | Methods; Data Synthesis  . |
| **Results** | | | |
| Study inclusion | 14 | This section should allow the reader to clearly follow how the included studies were identified and selected for inclusion in the review. There should be a narrative description of the process accompanied by a PRISMA flowchart; details to be reported include narrative summary of the numbers of studies identified, numbers screened, studies selected for retrieval and included/excluded and their reasons for exclusion, numbers appraised and included/excluded, and numbers included in the review. | Results; Study Inclusion, Figure 1. PRISMA Flow Diagram |
| Methodological quality | 15 | This section should focus on methodological quality as determined by the relevant critical appraisal instrument. There should be a separate narrative summary for the overall methodological quality of the quantitative (and quantitative component of mixed methods studies) and qualitative studies (and qualitative component of mixed methods studies), which can be supported by tables showing the results of the critical appraisal. | Results; Methodological Quality, Additional File 5 |
| Characteristics of included studies | 16 | This section of the results should include an overall description of the included studies (with reference to the table of included study characteristics in the appendices), with the main aim to provide some context to the results section and sufficient detail for the reader to confirm that the studies match the eligibility criteria for the review. | Results; Description of Included Studies, Table 2 |
| Findings of the review | 16 | Review findings are structured according to the phenomena of interest and should describe all the identified integrated finding(s), the categories that form them and the underpinning qualitative and/or qualitized data. Integrated findings should be presented with an explanatory statement that conveys the inclusive meaning of a group of similar categories (i.e. line of action statements). | Results; Categories, Table 3 Discussion; Summery of Main findings |
| Note | 17 | Please note: Due to the complexities associated with recommendations being derived from both streams of evidence and the impact of data transformation and/or integration on the grading process, an assessment of the certainty of the evidence using either the GRADE or ConQual approach is currently not recommended for JBI MMSR following either the integrated or segregated approach and requires further investigation. | Discussion; Strengths and Limitations |
| **Discussion** | | | |
| Discussion | 18 | This section should provide a detailed discussion of issues arising from the conduct of the review, as well as a discussion of the findings of the review and of the significance of the review findings in relation to practice and research. | Discussion; Summary of Main Findings; Integrated Finding 1; Integrated Finding 2; Implications for Practice; Implications for Research |
| **Conclusions and Recommendations** | | | |
| Conclusions | 19 | This section should include the overall conclusions of the review. The conclusions should provide direct answers to the review question(s). These conclusions should be based only on the results of the review and directly inferred from the review results. | Conclusions |
| Recommendations for practice | 20 | Include the recommendations for practice inferred from the results of the integration of the ‘qualitized’ data and qualitative data. | Discussion; Implications for Practice |
| Recommendations for research | 21 | Include the recommendations for future research inferred from the results of the integration of the ‘qualitized’ data and qualitative data, and issues and problems noted in the review process related to the search, selection of studies, critical appraisal, data extraction, and data synthesis. | Discussion; Implications for Research |
| **Conflicts and acknowledgements** | | | |
| Conflicts of interest | 22 | A statement which either declares the absence of any conflicts of interest or which describes a specified or potential conflict of interest should be made by the reviewers in this section | Conflict of Interest |
| Funding | 23 | Provide details regarding any sources of funding for the review project. The role of all funders in the review process, if any, should be explicitly described. If the review is funded, then any potential conflicts of interest or intellectual bias of the funders should be specified in the review. | Funding |
| Acknowledgements | 24 | Any acknowledgements should be made in this section e.g. sources of external funding or the contribution of colleagues or institutions | Acknowledgements |
