# Supplementary material for: Interventions to Prevent Potentially Avoidable Hospitalizations: A Mixed Methods Systematic Review
Source: Front Public Health. 2022 Jul 11;10:898359. doi: 10.3389/fpubh.2022.898359 (PMC9309492; doi:10.3389/fpubh.2022.898359)
Supplement: Supplementary file 3 [file Data_Sheet_3.docx]

**Additional File 3.** Overview of the Phases from Aim to Lines of Action

| **Phases of the mixed methods systematic review following a convergent integrative approach** | | | | | | | |
| --- | --- | --- | --- | --- | --- | --- | --- |
| Aim | To identify and synthesize evidence on interventions targeting avoidable hospitalizations from the perspectives of the citizens (age: ≥18 years) and the healthcare professionals to improve the preventive healthcare services. | | | | | | |
| **Methods** | | | | | | | |
| Review question | From the perspectives of the citizens (age: ≥18 years) and the healthcare professionals, which intervention components intend to prevent avoidable hospitalizations, focusing on why interventions work or not? | | | | | | |
| Inclusion criteria | Quantitative, qualitative and mixed methods studies published in English, Swedish, Norwegian or Danish. | | | | | | |
| Population | Citizens (age: ≥18 years)  Healthcare professionals from the primary and secondary healthcare sector | | | | | | |
| Phenomena of interest | Interventions targeting the prevention of avoidable hospitalizations, and why interventions work or not. | | | | | | |
| Context | Interventions performed in primary healthcare settings including home setting or community care setting; and interventions across primary and secondary healthcare settings, e.g., intermediate care.  Studies undertaken in developed countries with universal healthcare. | | | | | | |
| Search strategy | Databases used: Scopus, PubMed, The Cochrane Library, SveMed+, CINAHL and Embase.  Search terms and key words were adapted for each database by combining search terms related to: (1) avoidable admission, e.g., avoidable admission OR preventable admission, AND (2) the context; developed countries with universal health care, e.g. Austria OR Belgium OR Denmark. | | | | | | |
| Quality assessment | Independent reviewers critically appraised relevant articles prior to inclusion using JBI critical appraisal checklists. | | | | | | |
| Data extraction | Extracted data include county, aim, study design, population, phenomena of interest including intervention components, context and results of relevance to review questions. | | | | | | |
| Data synthesis | Quantitative data were transformed into textual descriptions or narrative interpretations, and then combined with the qualitative data. Assembled data were categorized and synthesized into integrated findings to produce line of action statements. | | | | | | |
| **Conducting the process** | | | | | | | |
| Process | Search (n=4322) | Study selection and critical appraisal | | Accepted methodological sound studies  (n=25) | Extracted findings (n=99) | Categorizing:  4 categories | Synthesizing:  2 integrated findings |
| **Integrated findings** | | | **Lines of action** | | | | |
| Integrated finding 1: Addressing individual needs through care continuity and coordination prevent avoidable hospitalizations | | | - Care practices should address both health-related issues and individual needs. - Clinical practice needs to be transformed to facilitate trustful relations between the citizen and healthcare professional, to allow healthcare professionals provide continuous and coordinated care, and to increase the involvement of the individual in preventive care practices. - Roles and responsibilities in multidisciplinary collaborations should be determined before initiation. | | | | |
| Integrated finding 2: Recognizing preventive care as an integrated part of the healthcare work to prevent avoidable hospitalizations | | | - Preventive care must be an integral part of the care work to ensure patient safety. - Healthcare administers and policymakers should support the preventive care by providing targeted educational material for healthcare professionals and simple web-based IT platforms for sharing information across healthcare settings. - Available tools need to take a broader perspective on individual health and functioning to prevent avoidable hospitalizations among those with complex care needs. | | | | |
