# Supplementary material for: Interventions to Prevent Potentially Avoidable Hospitalizations: A Mixed Methods Systematic Review
Source: Front Public Health. 2022 Jul 11;10:898359. doi: 10.3389/fpubh.2022.898359 (PMC9309492; doi:10.3389/fpubh.2022.898359)
Supplement: Supplementary file 4 [file Data_Sheet_4.docx]

**Additional File 4.** PubMed Search Strategy.

June 19 2019. N=864

[all fields] = Search in all fields

[MeSH] = Medical Subject Headings; controlled vocabulary thesaurus used for indexing articles in PubMed

[title] = Search in title

1. "avoidable admission" [all fields]
2. "avoidable admissions" [all fields]
3. "preventable admission" [all fields]
4. "preventable admissions" [all fields]
5. "inappropriate admission" [all fields]
6. "inappropriate admissions" [all fields]
7. "unnecessary admission" [all fields]
8. "unnecessary admissions" [all fields]
9. "ambulatory care sensitive" [all fields]
10. "avoidable hospitalization" [all fields]
11. "avoidable hospitalizations" [all fields]
12. "preventable hospitalization" [all fields]
13. "preventable hospitalizations" [all fields]
14. "inappropriate hospitalization" [all fields]
15. "inappropriate hospitalizations" [all fields]
16. "unnecessary hospitalization" [all fields]
17. "unnecessary hospitalizations" [all fields]
18. "avoidable hospital admission" [all fields]
19. "avoidable hospital admissions" [all fields]
20. "preventable hospital admission" [all fields]
21. "preventable hospital admissions" [all fields]
22. "inappropriate hospital admission" [all fields]
23. "inappropriate hospital admissions" [all fields]
24. "unnecessary hospital admission" [all fields]
25. "unnecessary hospital admissions" [all fields]
26. "avoidable hospitalisation" [all fields]
27. "avoidable hospitalisations" [all fields]
28. "preventable hospitalisation" [all fields]
29. "preventable hospitalisations" [all fields]
30. "inappropriate hospitalisation" [all fields]
31. "inappropriate hospitalisations" [all fields]
32. "unnecessary hospitalisation" [all fields]
33. "unnecessary hospitalisations"[all fields]
34. 1 OR 2 OR 3 OR 4 OR 5 OR 6 OR 7 OR 8 OR 9 OR 10 OR 11 OR 12 OR 13 OR 14 OR 15 OR 16 OR 17 OR 18 OR 19 OR 20 OR 21 OR 22 OR 23 OR 24 OR 25 OR 26 OR 27 OR 28 OR 29 OR 30 OR 31 OR 32 OR 33
35. Japan [all fields] [Mesh]
36. Canada [all fields] [Mesh]
37. Australia [all fields] [Mesh]
38. Switzerland [all fields] [Mesh]
39. Norway [all fields] [Mesh]
40. Iceland [all fields] [Mesh]
41. Slovenia [all fields] [Mesh]
42. Slovakia [all fields] [Mesh]
43. Romania [all fields] [Mesh]
44. Poland [all fields] [Mesh]
45. Malta [all fields] [Mesh]
46. Lithuania [all fields] [Mesh]
47. Latvia [all fields] [Mesh]
48. Hungary [all fields] [Mesh]
49. Estonia [all fields] [Mesh]
50. "Czech Republic" [Mesh]
51. "Czech Republic" [all fields]
52. Cyprus [all fields] [Mesh]
53. Croatia [all fields] [Mesh]
54. Bulgaria [all fields] [Mesh]
55. "Great Britain" [all fields]
56. Wales [all fields] [Mesh]
57. Scotland [all fields] [Mesh]
58. England [all fields] [Mesh]
59. "New Zealand" [Mesh]
60. "New Zealand" [all fields]
61. "United Kingdom" [Mesh]
62. "United Kingdom" [all fields]
63. Sweden [all fields] [Mesh]
64. Spain [all fields] [Mesh]
65. Portugal [all fields] [Mesh]
66. Holland [all fields]
67. Netherlands [all fields] [Mesh]
68. Luxembourg [all fields] [Mesh]
69. Italy [all fields] [Mesh]
70. Ireland [all fields] [Mesh]
71. Greece [all fields] [Mesh]
72. Germany [all fields] [Mesh]
73. France [all fields] [Mesh]
74. Finland [all fields] [Mesh]
75. Denmark [all fields] [Mesh]
76. Belgium [all fields] [Mesh]
77. Austria [all fields] [Mesh]
78. 35 OR 36 OR 37 OR 38 OR 39 OR 40 OR 41 OR 42 OR 43 OR 44 OR 45 OR 46 OR 47 OR 48 OR 49 OR 50 OR 51 OR 52 OR 53 OR 54 OR 55 OR 56 OR 57 OR 58 OR 59 OR 60 OR 61 OR 62 OR 63 OR 64 OR 65 OR 66 OR 67 OR 68 OR 69 OR 70 OR 71 OR 72 OR 73 OR 74 OR 75 OR 76 OR 77
79. Infant [title]
80. Infants [title]
81. Children [title]
82. 79 OR 80 OR 81
83. (34 AND 78) NOT 82
