# Supplementary material for: Interventions to Prevent Potentially Avoidable Hospitalizations: A Mixed Methods Systematic Review
Source: Front Public Health. 2022 Jul 11;10:898359. doi: 10.3389/fpubh.2022.898359 (PMC9309492; doi:10.3389/fpubh.2022.898359)
Supplement: Supplementary file 5 [file Data_Sheet_5.docx]

**Additional File 5.** Quality Appraisal Results.

| Critical appraisal results using the JBI Qualitative Critical Appraisal Checklist (and qualitative component of mixed methods studies) (Reviewers: CNL and MB) | | | | | | | | | | | |
| --- | --- | --- | --- | --- | --- | --- | --- | --- | --- | --- | --- |
| **Study** | **Q1** | **Q2*** | **Q3*** | **Q4*** | **Q5** | **Q6*** | **Q7*** | **Q8** | **Q9** | **Q10** | **Overall appraisal** |
| Cole M (1) | U | Y | U | U | Y | N | N | Y | U | Y | Exclusion |
| Dodd et al. (2) | Y | U | U | N | N | Y | N | N | Y | Y | Exclusion |
| Glasby et al. (3) | U | Y | Y | Y | Y | Y | Y | Y | Y | Y | Inclusion |
| Grant et al. (4) | U | Y | Y | Y | Y | Y | Y | Y | Y | Y | Inclusion |
| Jones et al. (5) | Y | Y | Y | U | U | Y | N | U | U | Y | Exclusion |
| Knight C & Dening KH (6) | U | U | U | U | U | U | N | N | N | U | Exclusion |
| Lee et al. (7) | U | U | U | U | U | N | N | Y | U | Y | Exclusion |
| Leighton et al. (8) | U | Y | Y | U | U | U | N | Y | Y | Y | Exclusion |
| Lhussier et al. (9) | Y | Y | Y | Y | Y | Y | Y | Y | Y | Y | Inclusion |
| Manning S (10) | Y | Y | Y | Y | Y | U | U | U | Y | Y | Exclusion |
| Millar et al. (11) | U | Y | Y | Y | Y | Y | Y | Y | Y | Y | Inclusion |
| Ploeg et al. (12) | U | Y | Y | Y | Y | Y | Y | Y | Y | Y | Inclusion |

Y=yes, N=no, U=unclear

*Criteria that must be met for articles to be included in final review

| Critical appraisal results using the JBI Critical Appraisal Checklist for Randomized Controlled Trials (Reviewers: CNL and AR) | | | | | | | | | | | | | | |
| --- | --- | --- | --- | --- | --- | --- | --- | --- | --- | --- | --- | --- | --- | --- |
| **Study** | **Q1** | **Q2** | **Q3** | **Q4** | **Q5** | **Q6** | **Q7** | **Q8** | **Q9** | **Q10*** | **Q11*** | **Q12*** | **Q13*** | **Overall appraisal** |
| Matzen et al. (13) | Y | Y | Y | N | N | U | N | Y | Y | Y | Y | Y | Y | Inclusion |

Y=yes, N=no, U=unclear

*Criteria that must be met for articles to be included in final review

| Critical appraisal results using the JBI Critical Appraisal Checklist for Quasi-Experimental Studies (Reviewers: CNL and MJJ) | | | | | | | | | | |
| --- | --- | --- | --- | --- | --- | --- | --- | --- | --- | --- |
| **Study** | **Q1*** | **Q2*** | **Q3** | **Q4** | **Q5** | **Q6** | **Q7** | **Q8*** | **Q9*** | **Overall appraisal** |
| Brownhill K. (14) | Y | U | U | N | Y | U | U | U | U | Exclusion |
| Freund et al. (15) | Y | Y | Y | Y | N | Y | Y | Y | Y | Inclusion |
| Garcia-Talvera et al. (16) | Y | Y | U | N | Y | N | Y | Y | N | Exclusion |
| Hullick et al. (17) | Y | Y | Y | Y | Y | N | Y | Y | Y | Inclusion |
| Islam MK & Kjerstad E (18) | Y | Y | Y | Y | Y | U | U | Y | Y | Inclusion |
| Jiwa et al. (19) | N | Y | Y | Y | N | U | U | U | U | Exclusion |
| Mahto et al. (20) | U | U | U | U | U | U | N | N | N | Exclusion |
| Mayhew L (21) | Y | Y | Y | N | Y | Y | Y | Y | Y | Inclusion |
| Stokes et al. (22) | Y | Y | U | Y | U | N | Y | Y | Y | Inclusion |
| Weatherall et al. (23) | Y | Y | U | Y | Y | U | N | Y | Y | Inclusion |
| Zhang et al. (24) | Y | Y | N | Y | Y | Y | Y | Y | Y | Inclusion |

Y=yes, N=no, U=unclear

*Criteria that must be met for articles to be included in final review

| Critical appraisal results using the JBI Critical Appraisal Checklist for Cohort Studies (Reviewers: CNL and AR) | | | | | | | | | | | | |
| --- | --- | --- | --- | --- | --- | --- | --- | --- | --- | --- | --- | --- |
| **Study** | **Q1** | **Q2** | **Q3*** | **Q4** | **Q5*** | **Q6** | **Q7*** | **Q8** | **Q9** | **Q10** | **Q11*** | **Overall appraisal** |
| Billot et al. (25) | Y | Y | Y | Y | Y | Y | Y | Y | Y | Y | Y | Inclusion |
| Hortelano et al. (26) | N | N | Y | N | N | N | Y | U | U | N | N | Exclusion |
| Iezzi et al. (27) | Y | Y | Y | Y | Y | Y | Y | U | N | U | Y | Inclusion |
| Manns et al. (28) | Y | Y | Y | Y | Y | Y | Y | U | U | U | Y | Inclusion |
| Seidu et al. (29) | U | Y | Y | Y | Y | Y | Y | Y | U | Y | Y | Inclusion |
| Wensing et al. (30) | Y | Y | Y | Y | Y | Y | Y | Y | Y | U | Y | Inclusion |
| Wensing et al. (31) | Y | Y | Y | Y | Y | Y | Y | Y | N | N | Y | Inclusion |

Y=yes, N=no, U=unclear

*Criteria that must be met for articles to be included in final review

| Critical appraisal results using the JBI Critical Appraisal Checklist for Case Series (Reviewers: CNL and MJJ) | | | | | | | | | | | |
| --- | --- | --- | --- | --- | --- | --- | --- | --- | --- | --- | --- |
| **Study** | **Q1*** | **Q2** | **Q3*** | **Q4*** | **Q5*** | **Q6** | **Q7** | **Q8*** | **Q9** | **Q10*** | **Overall appraisal** |
| Carlill et al. (32) | N | U | N | Y | N | N | N | N | U | N | Exclusion |
| Caughey et al. (33) | Y | Y | Y | N | N | Y | U | U | N | U | Exclusion |
| Fiorentini et al. (34) | Y | Y | Y | Y | Y | Y | N | Y | Y | Y | Inclusion |
| Graffy et al. (35) | Y | U | N | Y | U | Y | Y | U | Y | U | Exclusion |
| Ha et al. (36) | Y | Y | Y | Y | Y | Y | Y | Y | Y | Y | Inclusion |
| Kossovsky et al. (37) | N | N | N | N | N | U | U | Y | N | Y | Exclusion |
| Latif et al. (38) | Y | U | N | N | Y | Y | N | Y | U | U | Exclusion |
| Mayo A & Allen A (39) | N | U | U | Y | Y | N | N | U | Y | N | Exclusion |
| Menec et al. (40) | Y | Y | Y | Y | Y | Y | Y | Y | Y | Y | Inclusion |
| Patel et al. (41) | Y | Y | Y | Y | Y | N | N | Y | Y | Y | Inclusion |
| Petersen et al. (42) | N | U | U | U | N | N | N | N | N | N | Exclusion |
| Quinn D (43) | N | U | U | Y | U | N | N | N | U | U | Exclusion |
| Weiss et al. (44) | Y | N | Y | Y | Y | Y | Y | Y | N | Y | Inclusion |

Y=yes, N=no, U=unclear

*Criteria that must be met for articles to be included in final review

| Critical appraisal results using the JBI Critical Appraisal Checklist for Cross-sectional Studies (Reviewers: CNL and MJJ) | | | | | | | | | |
| --- | --- | --- | --- | --- | --- | --- | --- | --- | --- |
| **Study** | **Q1** | **Q2** | **Q3*** | **Q4** | **Q5** | **Q6*** | **Q7*** | **Q8*** | **Overall appraisal** |
| Barker et al. (45) | Y | Y | Y | Y | N | Y | Y | Y | Inclusion |

Y=yes, N=no, U=unclear

*Criteria that must be met for articles to be included in final review

**References**

1. Cole MS. Case Study: Realizing the Value of Nurse Practitioners in Long-Term Care. *Nurs Leadersh (Tor Ont)* (2017) **30**:39–44. doi:10.12927/cjnl.2017.25450

2. Dodd J, Taylor CE, Bunyan P, White PM, Thomas SM, Upton D. A service model for delivering care closer to home. *Prim Health Care Res Dev* (2011) **12**:95–111. doi:10.1017/S1463423610000356

3. Glasby J, Martin G, Regen E. Older people and the relationship between hospital services and intermediate care: Results from a national evaluation. *J Interprof Care* (2008) **22**:639–649. doi:10.1080/13561820802309729

4. Grant A, Dreischulte T, Guthrie B. Process evaluation of the data-driven quality improvement in primary care (DQIP) trial: active and less active ingredients of a multi-component complex intervention to reduce high-risk primary care prescribing. *Implement Sci* (2017) **12**:4. doi:10.1186/s13012-016-0531-2

5. Jones B, Hopkins G, Wherry SA, Lueck CJ, Das CP, Dugdale P. Evaluation of a Regional Australian Nurse-Led Parkinson’s Service Using the Context, Input, Process, and Product Evaluation Model. *Clin Nurse Spec* (2016) **30**:264–270. doi:10.1097/NUR.0000000000000232

6. Knight C, Dening KH. Management of long-term conditions and dementia: The role of the Admiral Nurse. *Br J Community Nurs* (2017) **22**:295–302. doi:10.12968/bjcn.2017.22.6.295

7. Lee G, Pickstone N, Facultad J, Titchener K. The future of community nursing: Hospital in the Home. *Br J Community Nurs* (2017) **22**:174–180. doi:10.12968/bjcn.2017.22.4.174

8. Leighton Y, Clegg A, Bee A. Evaluation of community matron services in a large metropolitan city in England. *Qual Prim Care* (2008) **16**:83–90.

9. Lhussier M, Dalkin S, Hetherington R. Community care for severely frail older people: Developing explanations of how, why and for whom it works. *Int J Older People Nurs* (2019) **14**: doi:10.1111/opn.12217

10. Manning SN. A multiple case study of patient journeys in Wales from A&E to a hospital ward or home. *Br J Community Nurs* (2016) **21**:509–517. doi:10.12968/bjcn.2016.21.10.509

11. Millar AN, Hughes CM, Ryan C. “It’s very complicated”: A qualitative study of medicines management in intermediate care facilities in Northern Ireland. *BMC Health Serv Res* (2015) **15**: doi:10.1186/s12913-015-0869-1

12. Ploeg J, Kaasalainen S, McAiney C, Martin-Misener R, Donald F, Wickson-Griffiths A, Carter N, Sangster-Gormley E, Schindel Martin L, Brazil K, et al. Resident and family perceptions of the nurse practitioner role in long term care settings: a qualitative descriptive study. *BMC Nurs* (2013) **12**: doi:10.1186/1472-6955-12-24

13. Matzen LE, Foged L, Pedersen P, Wengle K, Andersen-Ranberg K. Geriatrisk teambesøg kan forebygge indlæggelse af henviste patienter men er et tidskrævende tilbud - En randomiseret undersøgelse [Geriatric home visits can prevent hospitalisation of subacute patients but is time-consuming - a randomised study]. *Ugeskr laeger [Dan Med J]* (2007) **169**:2113–2118.

14. Brownhill KM. Training in care homes to reduce avoidable harm. *Nurs Times* (2013) **109**:20–22.

15. Freund T, Peters-Klimm F, Boyd CM, Mahler C, Gensichen J, Erler A, Beyer M, Gondan M, Rochon J, Gerlach FM, et al. Medical assistant-based care management for high-risk patients in small primary care practices: A cluster randomized clinical trial. *Ann Intern Med* (2016) **164**:323–330. doi:10.7326/M14-2403

16. García-Talavera Espín NV, López-Ruiz A, Nuñez Sánchez MÁ, Meoro Avilés A, Sánchez Cañizares C, Romero López-Reinoso H, López Olivar MD, Lapaz Jorge MÁ, Guirao Sastre JM, San Eustaquio Tudanca F, et al. How to reduce avoidable admissions due to acute diabetes complications? Interrelation between primary and specialized attention in a diabetes unit. *Nutr Hosp* (2012) **27**:2079–2088. doi:10.3305/nh.2012.27.6.6151

17. Hullick C, Conway J, Higgins I, Hewitt J, Dilworth S, Holliday E, Attia J. Emergency department transfers and hospital admissions from residential aged care facilities: A controlled pre-post design study. *BMC Geriatr* (2016) **16**:1–10. doi:10.1186/s12877-016-0279-1

18. Islam MK, Kjerstad E. Co-ordination of health care: the case of hospital emergency admissions. *Eur J Heal Econ* (2019) **20**:525–541. doi:10.1007/s10198-018-1015-x

19. Jiwa M, Gerrish K, Gibson A, Scott H. Preventing avoidable hospital admission of older people. *Br J Community Nurs* (2002) **7**:426–431.

20. Mahto R, Venugopal H, Vibhuti VS, Mukherjee A, Cherukuri V, Healey B, Baskar V, Buch HN, Singh BM. The effectiveness of a hospital diabetes outreach service in supporting care for acutely admitted patients with diabetes. *QJM* (2009) **102**:203–207. doi:10.1093/qjmed/hcn174

21. Mayhew L. On the effectiveness of care co-ordination services aimed at preventing hospital admissions and emergency attendances. *Health Care Manag Sci* (2009) **12**:269–284. doi:10.1007/s10729-008-9092-5

22. Stokes J, Kristensen SR, Checkland K, Bower P. Effectiveness of multidisciplinary team case management: Difference-indifferences analysis. *BMJ Open* (2016) **6**: doi:10.1136/bmjopen-2015-010468

23. Weatherall CD, Hansen AT, Nicholson S. The effect of assigning dedicated general practitioners to nursing homes. *Health Serv Res* (2019) **54**:547–554. doi:10.1111/1475-6773.13112

24. Zhang J, Donald M, Baxter KA, Ware RS, Burridge L, Russell AW, Jackson CL. Impact of an integrated model of care on potentially preventable hospitalizations for people with Type 2 diabetes mellitus. *Diabet Med* (2015) **32**:872–880. doi:10.1111/dme.12705

25. Billot L, Corcoran K, McDonald A, Powell-Davies G, Feyer A-M. Impact Evaluation of a System-Wide Chronic Disease Management Program on Health Service Utilisation: A Propensity-Matched Cohort Study. *PLoS Med* (2016) **13**: doi:10.1371/journal.pmed.1002035

26. Hortelano CME, Rodero FG, Muñoz JE, Santaleocadia CB, Canuto MM, Hidalgo AM, Rufete AM, Aguado IH. Impact of a day-care center on the appropriateness of hospital admissions and frequency of hospitalization in patients with HIV infection. *Br J Clin Gov* (2001) **6**:102–108. doi:10.1108/14664100110397214

27. Iezzi E, Lippi Bruni M, Ugolini C. The role of GP’s compensation schemes in diabetes care: Evidence from panel data. *J Health Econ* (2014) **34**:104–120. doi:10.1016/j.jhealeco.2014.01.002

28. Manns BJ, Tonelli M, Zhang J, Campbell DJT, Sargious P, Ayyalasomayajula B, Clement F, Johnson JA, Laupacis A, Lewanczuk R, et al. Enrolment in primary care networks: Impact on outcomes and processes of care for patients with diabetes. *CMAJ* (2012) **184**:E144–E152. doi:10.1503/cmaj.110755

29. Seidu S, Bodicoat DH, Davies MJ, Daly H, Stribling B, Farooqi A, Brady EM, Khunti K. Evaluating the impact of an enhanced primary care diabetes service on diabetes outcomes: A before–after study. *Prim Care Diabetes* (2017) **11**:171–177. doi:10.1016/j.pcd.2016.09.005

30. Wensing M, Szecsenyi J, Stock C, Kaufmann Kolle P, Laux G. Evaluation of a program to strengthen general practice care for patients with chronic disease in Germany. *BMC Health Serv Res* (2017) **17**:1–7. doi:10.1186/s12913-017-2000-2

31. Wensing M, Kolle PK, Szecsenyi J, Stock C, Laux G. Effects of a program to strengthen general practice care on hospitalisation rates: a comparative observational study. *Scand J Prim Health Care* (2018) **36**:109–114. doi:10.1080/02813432.2018.1459429

32. Carlill G, Gash E, Hawkins G. Preventing unnecessary hospital admissions: An occupational therapy and social work service in an accident and emergency department. *Br J Occup Ther* (2002) **65**:440–445. doi:10.1177/030802260206501002

33. Caughey GE, Hillen JB, Bacon S, Bullock N, Bullock V, Kalisch Ellett L. Implementation of medication-related indicators of potentially preventable hospitalizations in a national chronic disease management program for older patients with multimorbidity. *Int J Qual Heal Care* (2019) **31**:133–139. doi:10.1093/intqhc/mzy133

34. Fiorentini G, Iezzi E, Bruni ML, Ugolini C. Incentives in primary care and their impact on potentially avoidable hospital admissions. *Eur J Heal Econ* (2011) **12**:297–309. doi:10.1007/s10198-010-0230-x

35. Graffy J, Grande M, Campbell J. Case management for elderly patients at risk of hospital admission: A team approach. *Prim Heal Care Res Dev* (2008) **9**:7–13. doi:10.1017/S1463423607000564

36. Ha NT, Harris M, Preen D, Robinson S, Moorin R. A time-duration measure of continuity of care to optimise utilisation of primary health care: a threshold effects approach among people with diabetes. *BMC Health Serv Res* (2019) **19**:276. doi:10.1186/s12913-019-4099-9

37. Kossovsky MP, Chopard P, Bolla F, Sarasin FP, Louis-Simonet M, Allaz A-F, Perneger T V, Gaspoz J-M. Evaluation of quality improvement interventions to reduce inapproapriate hospital use. *Int J Qual Heal Care* (2002) **14**:227–232. doi:10.1093/oxfordjournals.intqhc.a002614

38. Latif A, Mandane B, Anderson E, Barraclough C, Travis S. Optimizing medicine use for people who are homebound: an evaluation of a pilot domiciliary Medicine Use Review (dMUR) service in England. *Integr Pharm Res Pract* (2018) **Volume 7**:33–40. doi:10.2147/IPRP.S160149

39. Mayo A, Allen A. Reducing admissions with social enterprises. *Emerg Nurse* (2010) **18**:14–17. doi:10.7748/en2010.07.18.4.14.c7901

40. Menec VH, Sirski M, Attawar D, Katz A. Does continuity of care with a family physician reduce hospitalizations among older adults? *J Heal Serv Res Policy* (2006) **11**:196–201. doi:10.1258/135581906778476562

41. Patel R, Clancy R, Crowther E, Vannahme M, Pullyblank A. A rectal bleeding algorithm can successfully reduce emergency admissions. *Color Dis* (2014) **16**:377–381. doi:10.1111/codi.12524

42. Petersen H, Melton R, Sejtved B, S van der M. Helping patients avoid admission to hospital. *Sygeplejersken / Danish J Nurs* (2009) **109**:52–55.

43. Quinn D. A collaborative care pathway to reduce multiple sclerosis admissions to secondary care. *Br J Neurosci Nurs* (2011) **7**:497–499.

44. Weiss N, Courjon J, Pradier C, Caisso C, Mondain V, Roger P-M, Demonchy E. Fast track consultation in the infectious diseases department of a French university hospital: evaluation of the service delivered to the general practitioner. *Infect Dis (Auckl)* (2018) **50**:119–124. doi:10.1080/23744235.2017.1366043

45. Barker I, Steventon A, Deeny SR. Association between continuity of care in general practice and hospital admissions for ambulatory care sensitive conditions: cross sectional study of routinely collected, person level data. *BMJ* (2017) **356**: doi:10.1136/bmj.j84
